# Activation of gut FXR improves the metabolism of bile acids, intestinal barrier, and microbiota under cholestatic condition caused by GCDCA in mice

**DOI:** 10.1128/spectrum.03150-24

**Published:** 2025-02-21

**Authors:** Xing-Ming Xie, Bang-Yan Zhang, Shu Feng, Zi-Jun Fan, Guo-Ying Wang

**Affiliations:** 1 Guizhou Institute of Precision Medicine, The Affiliated Hospital of Guizhou Medical University, Guiyang, Guizhou, China; 2 Key Laboratory of Hepatobiliary and Pancreatic Diseases Treatment and Bioinformatics Research, Guizhou Medical University, Guiyang, Guizhou, China; 3 Department of Hepatobiliary Surgery, The First Affiliated Hospital of Guangzhou Medical University, Guangzhou, Guangdong, China; 4 Department of Gastrointestinal Surgery, The Third Affiliated Hospital of Zunyi Medical University (The First People’s Hospital of Zunyi), Zunyi, Guizhou, China; 5 Department of Respiratory and Critical Care Medicine, Guizhou Provincial People’s Hospital, Guiyang, Guizhou, China; 6 Key Laboratory of Pulmonary Immune Diseases, National Health Commission, Guiyang, Guizhou, China; 7 Department of Medical Examination Center, The Affiliated Hospital of Guizhou Medical University, Guiyang, Guizhou, USA; 8 The First Clinical School of Medicine, Guangzhou Medical University, Guangzhou, Guangdong, China; Lerner Research Institute, Cleveland, Ohio, USA

**Keywords:** cholestasis, glycochenodeoxycholate, GW4064, bile acid, gut microbiota

## Abstract

Abnormal bile acid (BA) metabolism is involved in liver fibrosis. In a previous study, we discovered that the hydrophobic BA glycochenodeoxycholate (GCDCA) induced liver fibrosis and that GW4064, an agonist of farnesoid X receptor (FXR), alleviated liver fibrosis caused by GCDCA. However, the impacts of GCDCA on liver BAs, gut BAs, the intestinal barrier, and the gut microbiota are unclear, and obtaining this information would provide additional information into the role of GCDCA in the development of liver fibrosis. In the present study, ultra-performance liquid chromatography‒tandem mass spectrometry revealed that mice administered GCDCA by gavage had higher levels of total and primary liver BAs than those in the control group, and a significant reduction in primary liver BAs was observed in the GCDCA + GW4064 group compared with those in the GCDCA group. Compared with those in the control group, the mice administered GCDCA by gavage had greater levels of total and primary BAs in the gut, especially T-alpha-MCA and T-beta-MCA, and no significant differences in the terminal ileum were observed between the GCDCA and GCDCA + GW4064 groups. Immunohistochemistry indicated that GCDCA administration inhibited gut FXR and FGF15 expression, whereas GW4064 activated gut FXR and promoted FGF15 expression. Moreover, immunohistochemistry revealed that GCDCA administration decreased mucin2, claudin-1, occludin, and ZO-1 expression, whereas GW4064 restored their expression. 16S rDNA sequencing revealed that the alpha diversity of the microbiota did not significantly differ among the three groups, but differences in the beta diversity of the microbiota were observed among the three groups. At the phylum level, GCDCA significantly disturbed the gut microbiota, as indicated by reductions in Desulfobacterota, Bacteroidota, and Actinobacteria in the GCDCA group compared with those in the control group. However, significantly increased abundances of Proteobacteria, Cyanobacteria, and Patescibacteria were noted in the GCDCA group compared with the control group. GW4064 administration significantly improved the microbiota structure at the phylum level. The efficacy of GW4064 was also observed at the genus level. Correlation analyses revealed fewer relationships between the gut microbiota and gut BAs, whereas the gut microbiota was more closely related to liver BAs in the GCDCA and GW4064 intervention groups. Together, GCDCA induced cholestasis and disturbed BA metabolism in the gut and liver, as well as the intestinal barrier and structure of the gut microbiota. Activation of gut FXR improved intestinal barrier injury and alleviated BA metabolism dysfunction and dysbacteriosis caused by GCDCA under cholestatic conditions.

**IMPORTANCE:**

Glycochenodeoxycholate (GCDCA) is a hydrophobic bile acid (BA) in humans and is highly increased in the serum and stool of liver fibrosis patients. However, the effects of GCDCA were not comprehensively investigated in the process of liver bile acid metabolism, gut microbiota, and intestinal barrier. It was reported that GCDCA can promote liver fibrosis via the NOD-like receptor family pyrin domain containing 3 (NLRP3) inflammasome pathway in mice, and gut farnesoid X receptor activation alleviated the fibrosis caused by GCDCA in our previous study. Gut microbiota is also responsible for BA metabolism; meanwhile, BA metabolism may also exert an effect on the intestinal barrier. Nowadays, the comprehensive understanding of gut microbiota and intestinal barrier in relation to BA disorder was still insufficient. Current study further investigated the role of GCDCA in BA metabolism, gut microbiota, and intestinal barrier to help understand the effects of GCDCA in liver fibrosis, which may provide intervention methods for liver fibrosis caused by dysregulation of BA metabolism.

## INTRODUCTION

The accumulation of hydrophobic bile acids (BAs) in cholestasis results in hepatocyte injury and fibrosis/cirrhosis (1–3). BAs are produced in hepatocytes, discharged into the gut via the bile duct, and reabsorbed in the gut. Moreover, gut BAs also regulate BA production in the liver through the activity of farnesoid X receptor (FXR) (4, 5), which is widely expressed in the ileum (6). BAs can impact bile-associated bacteria to alter the composition of the gut microbiota (7, 8). In contrast, gut bacteria also catalyze the conversion of primary BAs to secondary BAs, which increases the diversity of the BA pool (9). Therefore, the relationships among BAs, FXR, and the gut microbiota strongly influence liver fibrosis.

Glycochenodeoxycholate (GCDCA) is a hydrophobic BA in humans and is highly increased in the serum of patients with liver fibrosis (10, 11). In addition, GCDCA levels in the stool of nonobese patients markedly increase with worsening fibrosis severity (12). Hohenester et al. (13) reported that GCDCA increases the toxicity and hydrophobicity of the bile salt pool, which results in liver fibrosis in mice. Our previous study revealed that GCDCA promotes liver fibrosis via the NLRP3 inflammasome pathway in mice and that GW4064 alleviated the fibrosis caused by GCDCA (14). However, the impacts of GCDCA on gut bacteria, the intestinal barrier, gut BAs, and liver BAs remain unclear, and addressing these research gaps may help to elucidate the role of GCDCA in liver fibrosis and reveal potential interventions for liver fibrosis.

In the present work, mice were administered GCDCA to explore its impacts on the gut microbiota, intestinal barrier, gut BAs, and liver BAs. GW4064 is widely used as an agonist of FXR (15–17). GW4064 was used to determine whether it acts as an antagonist to GCDCA, affecting the gut microbiota, intestinal barrier, gut BAs, and liver BAs. The findings from this study are expected to provide some insights into liver fibrosis progression and treatment.

## MATERIALS AND METHODS

### Animal experiments

C57BL/6J male mice at 7 weeks of age were used for the intervention in accordance with our previous study (14). The mice were divided into three groups: the control group (10% dimethyl sulfoxide (DMSO) + 40% PEG300 + 5% Tween-80 + 45% saline; *n* = 5), the GCDCA gavage group (GCDCA powder dissolved in 10% DMSO, 40% PEG300, 5% Tween-80, and 45% saline; a dose of 1,000 mg/kg body weight of GCDCA was administered via a specific gavage needle; *n* = 4), and the GCDCA + GW4064 gavage group (GW4064 [30 mg/kg body weight] + GCDCA [1,000 mg/kg body weight], the dose of GW4064 was the same as that of GCDCA; *n* = 5). All the mice were tested three times a week for 12 weeks. All the mice were housed under a 12 h light/dark cycle at 20°C–22°C and 45 ± 5% humidity in a specific pathogen-free environment.

### Targeted metabolomics for BAs

The liver tissues and terminal ileum contents were collected for identification of the BA profiles using ultra-performance liquid chromatography‒tandem mass spectrometry (UPLC‒MS/MS) by LC-Bio Technology Co., Ltd. (Hangzhou, China). Then, 50 mg of the sample was placed into a 1.5 mL Eppendorf (EP) tube (on ice), and 0.5 mL of 80% methanol in water (precooled at −20°C) was added for protein precipitation. The sample was subsequently ground in a freeze-grinder for 3 minutes (50 Hz, 4°C), shaken, extracted in a multifunctional mixer for 20 minutes, and centrifuged (4°C, 20,000 rcf) for 15 minutes. The supernatant was transferred to a new 1.5 mL EP tube and centrifuged (4°C, 20,000 rcf) for 15 minutes. Finally, 200 µL of solution was transferred into an injection bottle for LC‒MS/MS analysis.

### Standard solution preparation

The standard substances were dissolved or diluted and mixed to obtain a 2 µg/mL mixed standard stock solution. The mixed standard stock solution was diluted stepwise to prepare standard solutions of different concentrations for standard curve generation.

### UPLC‒MS/MS analysis

A Vanquish liquid-phase system (Thermo) equipped with an Agilent Eclipse Plus C18 RRHD column (3.0 * 150 mm, 1.8 µm) was used for separation. Mobile phase A was a 10 mmol/L ammonium acetate aqueous solution, and mobile phase B was a methanol-acetonitrile solution (volume ratio = 1:1). The column temperature was set to 40°C, and the automatic sampler temperature was set to 4°C. The injection volume was 5 µL. The flow rate was 0.45 µL/min. The mobile phase elution gradient was as follows: 0–6 minutes, 40%–50% B; 6–8 minutes, 50% B; 8–11.5 minutes, 50%–60% B; 11.5–12 minutes, 60% B; 12–15.5 minutes, 60%–90% B; 15.5–17.5 minutes, 90%–98% B; 17.5–18 minutes, 98%; 18–18.1 minutes, 98–40% B; and 18.1–21 minutes, 40% B.

A TSQ Altis triple quadrupole mass spectrometer (Thermo) with an electrospray ionization (ESI) interface was used. The typical ion source parameters were as follows: negative ion voltage = 3,000 V, sheath gas = 20 Arb, collision gas = 10 Arb, auxiliary gas = 0 Arb, evaporation temperature = 350°C, and ion transfer tube temperature = 350°C. Flow injection analysis was conducted by injecting the standard solution of a single analyte into the ESI source of the mass spectrometer to optimize the multiple reaction monitoring (MRM) parameters of each target analyte. During the optimization process for each compound, the Q1/Q3 ratio with the highest response was selected as the quantitative ion, and the others were selected as the qualitative ions. Thermo Scientific Xcalibur (version 4.4) software was used for MRM data collection and processing (Supplementary S1).

#### Calculation formula


$$c_{M}\left[ng∙g^{-1}\right]=\frac{c_{F}\left[ng∙{mL}^{-1}\right]∙V_{F}[mL]∙CF}{m_{s}[g]}$$

The concentration of *c*_*F*_ (ng/mL) was calculated using Thermo Scientific Xcalibur software. The compound concentration (*c*_*M*_, ng/g) was calculated as *c*_*F*_ multiplied by the constant volume (*V*_*F*_, mL), and the dilution factor (*CF*) was divided by the sample weight (*m*_S_, g).

### Microbial DNA extraction and 16S rRNA sequencing

#### DNA extraction

A total of 250 mg of each sample of terminal ileum content was subjected to microbial DNA extraction using the Magnetic Soil and Stool DNA Kit (DP712, TIANGEN) according to the manufacturer’s instruction manual. The total DNA was eluted in 50 µL of elution buffer and stored at −80°C until PCR analysis.

### PCR amplification and 16S rDNA sequencing

The 341 forward primer (5′-CCTACGGGNGGCWGCAG-3) and the 805 reverse primer (5′-GACTACHVGGGTATCTAATCC-3) were used for prokaryotic 16S rRNA gene amplification of the V3-V4 region, and the 5´ ends of the primers were tagged with specific barcodes. The total volume of the reaction mixture was 25 µL, which consisted of 50 ng of template DNA, 12.5 µL of PCR Premix, 2.5 µL of each primer, and PCR-grade water. The PCR procedure involved the following steps: (i) initial denaturation at 98°C for 30 seconds; (ii) 32 cycles of denaturation at 98°C for 10 seconds, annealing at 54°C for 30 seconds, and extension at 72°C for 45 seconds; and (iii) a final extension at 72°C for 10 minutes.

The PCR products were confirmed with 2% agarose gel electrophoresis. Throughout the DNA extraction process, ultrapure water, instead of sample mixture, was used to exclude the possibility of false-positive PCR results as a negative control. The PCR products were purified with AMPure XT beads (Beckman Coulter Genomics, Danvers, MA, USA) and quantified with a Qubit fluorometer (Invitrogen, USA). The amplicon pools were prepared for sequencing, and the size and quantity of the amplicon library were assessed on an Agilent 2100 Bioanalyzer (Agilent, USA) and with a Library Quantification Kit for Illumina (Kapa Biosciences, Woburn, MA, USA), respectively. The libraries were sequenced on a NovaSeq PE250 platform by LC-Bio Technology Co., Ltd. (Hangzhou, China).

### Immunohistochemical staining

The liver and terminal ileum tissue sections were first deparaffinized and rehydrated. Antigen retrieval was then performed, followed by blocking of nonspecific binding sites with 5% bovine serum albumin (BSA) for 20 minutes. The sections were incubated overnight at 4°C with primary antibodies against FXR (1:200, 25055-1-AP, Proteintech), FGF15 (1:200, PK15481, ABmart), mucin2 (1:1,000, GB11344, Servicebio), claudin-1 (1:1,000, GB15032, Servicebio), occludin (1:500, GB111401, Servicebio), and ZO-1 (1:1,000, GB115686, Servicebio). Afterward, the samples were incubated in secondary antibody at room temperature for 60 minutes. Finally, the sections were examined under a microscope after diaminobenzidine (DAB) and hematoxylin staining.

### Statistical analysis

#### Targeted metabolomics data analysis

First, the normality of the distribution of the BA concentrations was determined by the Kolmogorov-Smirnov test. Normally distributed continuous data were assessed with Student’s *t*-test for two groups or one-way analysis of variance for three groups. Continuous data that were not normally distributed were analyzed with the Mann-Whitney U-test for two groups or the Kruskal-Wallis test for three groups. A *P*-value <0.05 was considered to indicate statistical significance.

### Sequencing data analysis

Cutadapt (v.1.9) was used to remove the sequencing primer from the demultiplexed raw sequences. Paired-end reads were merged using FLASH (v.1.2.8). The low-quality reads (quality scores <20), short reads (<100 bp), and reads containing more than 5% “N” records were trimmed using the sliding window algorithm to obtain high-quality clean tags in fqtrim (v.0.94). Chimeric sequences were filtered using Vsearch software (v.2.3.4). After denoising for amplicon sequence variations (ASVs) using DADA2, the ASV feature table and feature sequence were obtained. BLAST was used for sequence alignment, and the feature sequences were annotated with the SILVA and NT-16S databases for each representative sequence. Alpha diversity was used to analyze the complexity of species diversity in a sample on the basis of three indices, namely the Chao1, Shannon, and Simpson indices. All the indices for the samples were calculated with QIIME2. The beta diversity was calculated with QIIME2, and the graphs were drawn with the R package. On the basis of the ASV abundance table, principal component analysis (PCA) was conducted. Principal coordinate analysis (PCoA) and nonmetric multidimensional scaling (NMDS) based on the Bray-Curtis distance were performed. Finally, the abundance of species at each level in each sample was calculated on the basis of the ASV abundance table. The linear discriminant analysis (LDA) effect size (LEfSe, LDA ≥3.0, *P*-value <0.05) was calculated using nsegata-Lefer. The R package (v.3.5.2) was used for image production.

### Spearman correlation analysis

The correlations between the abundances of gut microbes and BAs were determined and visualized using OmicStudio Cloud (https://www.omicstudio.cn/). A *P*-value <0.05 was considered to indicate statistical significance.

## RESULTS

Our previous study revealed that GCDCA promotes liver fibrosis via the NLRP3 inflammasome pathway in mice and that GW4064 alleviates the fibrosis caused by GCDCA (14). The current study further investigated the role of GCDCA in BA metabolism, the intestinal barrier, and the gut microbiota to help elucidate the role of GCDCA in liver fibrosis.

### The gut FXR agonist GW4064 alleviated cholestatic conditions induced by GCDCA

**Fig 1:**
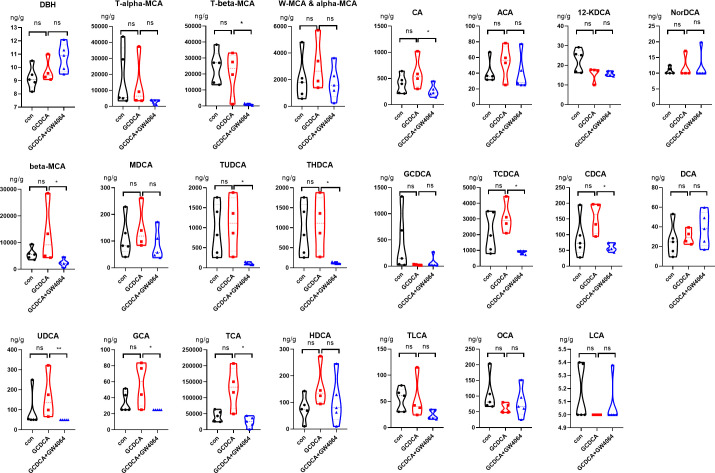
The bile acid profile of liver.

The BA profile of the liver was detected (Fig. 1). Compared with those in the control group, the levels of beta-muricholic acid (beta-MCA), murideoxycholic acid (MDCA), tauroursodeoxycholic acid (TUDCA), taurohyodeoxycholic acid (THDCA), ursodeoxycholic acid (UDCA), glycocholic acid (GCA), taurocholic acid (TCA), hyodeoxycholic acid (HDCA), cholic acid (CA), allocholic acid (ACA), taurochenodeoxycholic acid (TCDCA) and chenodeoxycholic acid (CDCA) were greater in the GCDCA group, but the differences were not significant. In contrast, treatment with GW4064, an agonist of FXR, markedly reduced the concentrations of T-beta-MCA, beta-MCA, TUDCA, THDCA, UDCA, GCA, TCA, CA, TCDCA, and CDCA, which were strongly induced by GCDCA (Fig. 1; Supplementary S2).

**Fig 2:**
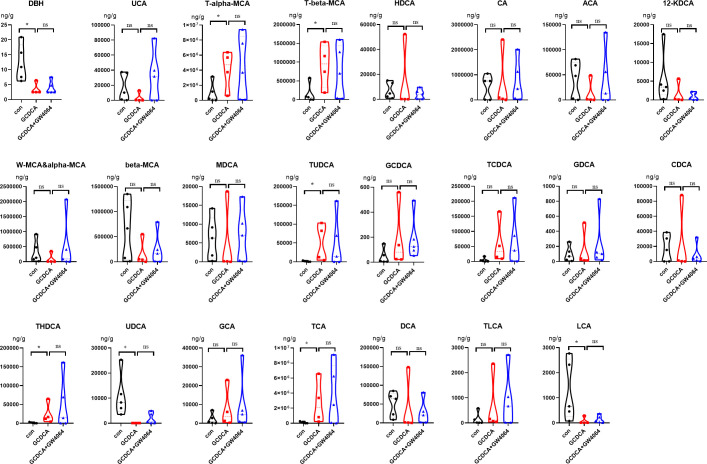
The bile acid profile of the terminal ileum.

The BA levels in the terminal ileum contents were also determined. The levels of T-alpha-MCA, T-beta-MCA, TUDCA, THDCA, and TCA were greater in the GCDCA group than in the control group. The levels of hydroguinonecarboxylicacid (DBH), UDCA, and lithocholic acid (LCA) were lower in the GCDCA group than in the control group. The differences in the BA levels between the GCDCA group and the GCDCA + GW4064 group were not significant (Fig. 2; Supplementary S3).

**Fig 3:**
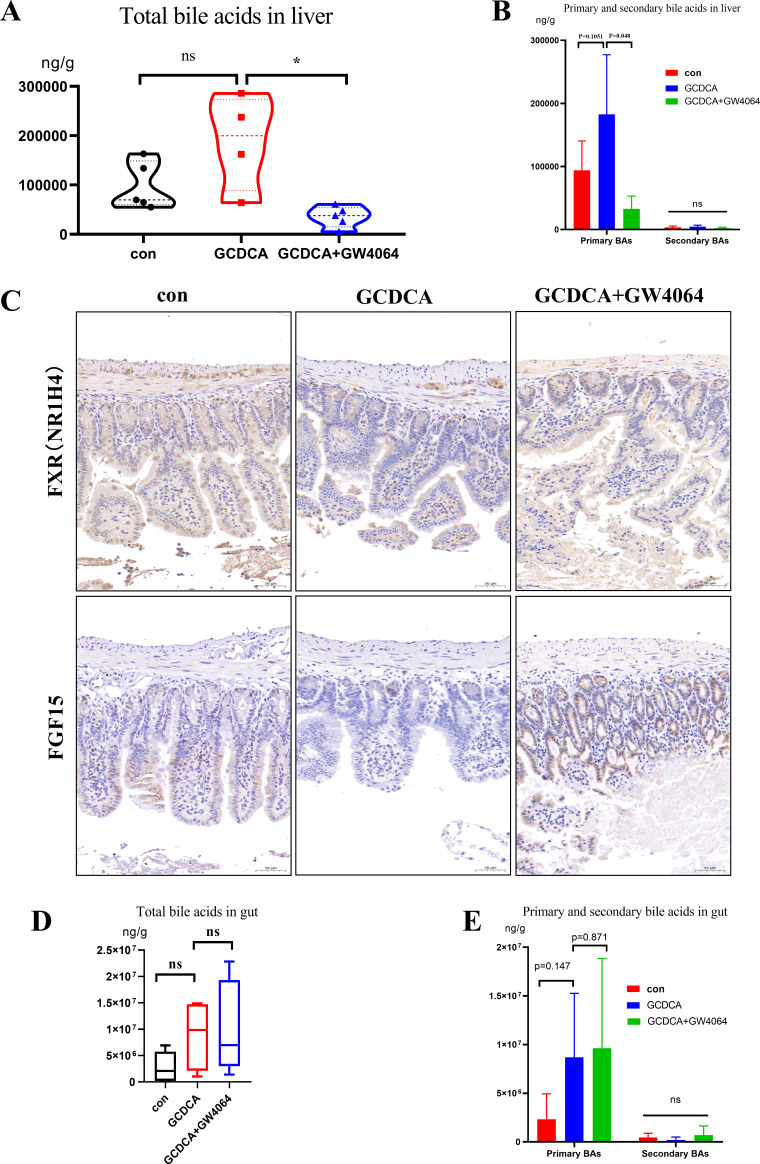
The concentration of bile acids in liver and terminal ileum, and activation of gut FXR by GW4064. (**A**) The concentration of total bile acids in liver. (**B**) The concentration of primary bile acids and secondary bile acids in liver. (**C**) GCDCA downregulated FXR and FGF15 expression in gut, and GW4064 activated their expression. (**D**) The concentration of total bile acids in terminal ileum. (**E**) The concentration of primary bile acids and secondary bile acids in the terminal ileum.

Compared with the control group, the GCDCA group presented higher total BA levels in the liver (187468.62 ± 96522.81 ng/g vs 97401.77 ± 48151.23 ng/g, *P* = 0.1082), and GW4064 markedly reduced the total BA levels in the liver compared with the GCDCA group (35316.63 ± 21224.21 ng/g vs 187468.62 ± 96522.81 ng/g, *P* = 0.048) (Fig. 3A). In addition, the GCDCA group presented a trend toward increased levels of primary BAs in the liver compared with the control group (182730.65 ± 94457.22 ng/g vs 93971.6 ± 46420.51 ng/g, *P* = 0.1051), and the GCDCA + GW4064 group presented lower liver primary BA levels than did the GCDCA group (33000.58 ± 20062.75 ng/g vs 182730.65 ± 94457.22 ng/g, *P* = 0.048) (Fig. 3B). The levels of total secondary bile acids in the liver did not differ among the three groups. In the terminal ileum, FXR activation promotes the expression of fibroblast growth factor 15 (mouse), which reaches the liver through the portal vein and inhibits the expression of *Cyp7a1*, attenuating BA production. The immunohistochemistry results indicated that GCDCA administration inhibited gut FXR and FGF15 expression, whereas GW4064 restored their expression (Fig. 3C). These results indicated that the administration of GCDCA induced cholestasis, whereas GW4064 alleviated cholestasis in the liver.

Compared with the control group, the GCDCA group presented greater total bile acid levels in the gut (8886052.41 ± 6776545.86 ng/g vs 2783280.64 ± 2937875.84 ng/g, *P* = 0.17), and there was no marked reduction in total BA levels in the gut in the GCDCA + GW4064 group compared with the GCDCA group (10304124.67 ± 8805289.13 ng/g vs 8886052.41 ± 6776545.86 ng/g, *P* = 0.793) (Fig. 3D). In addition, the GCDCA group presented increasing trends in the gut primary BA levels compared with those of the control group (8696497.00 ± 6573145.83 ng/g vs 2329070.26 ± 2610175.75 ng/g, *P* = 0.147), and no reduction in the levels of primary BAs was detected in the GCDCA + GW4064 group compared with those of the GCDCA group (9622555.02 ± 9213349.57 ng/g vs 8696497.00 ± 6573145.83 ng/g, *P* = 0.871) (Fig. 3E). The gut primary BA levels in the control group, GCDCA group, and GCDCA + GW4064 group were 2329070.26 ± 2610175.75 ng/g, 8696497.00 ± 6573145.83 ng/g, and 9622555.02 ± 9213349.57 ng/g, respectively. Compared with those in the GCDCA group, the gut primary BA levels in the GCDCA + GW4064 group tended to increase. These findings indicated that GCDCA administration increased the bile content in the gut, whereas GW4064 administration potentially induces bile discharge into the gut.

### The gut FXR agonist GW4064 improved the disorder of the gut microbiota induced by GCDCA

**Fig 4:**
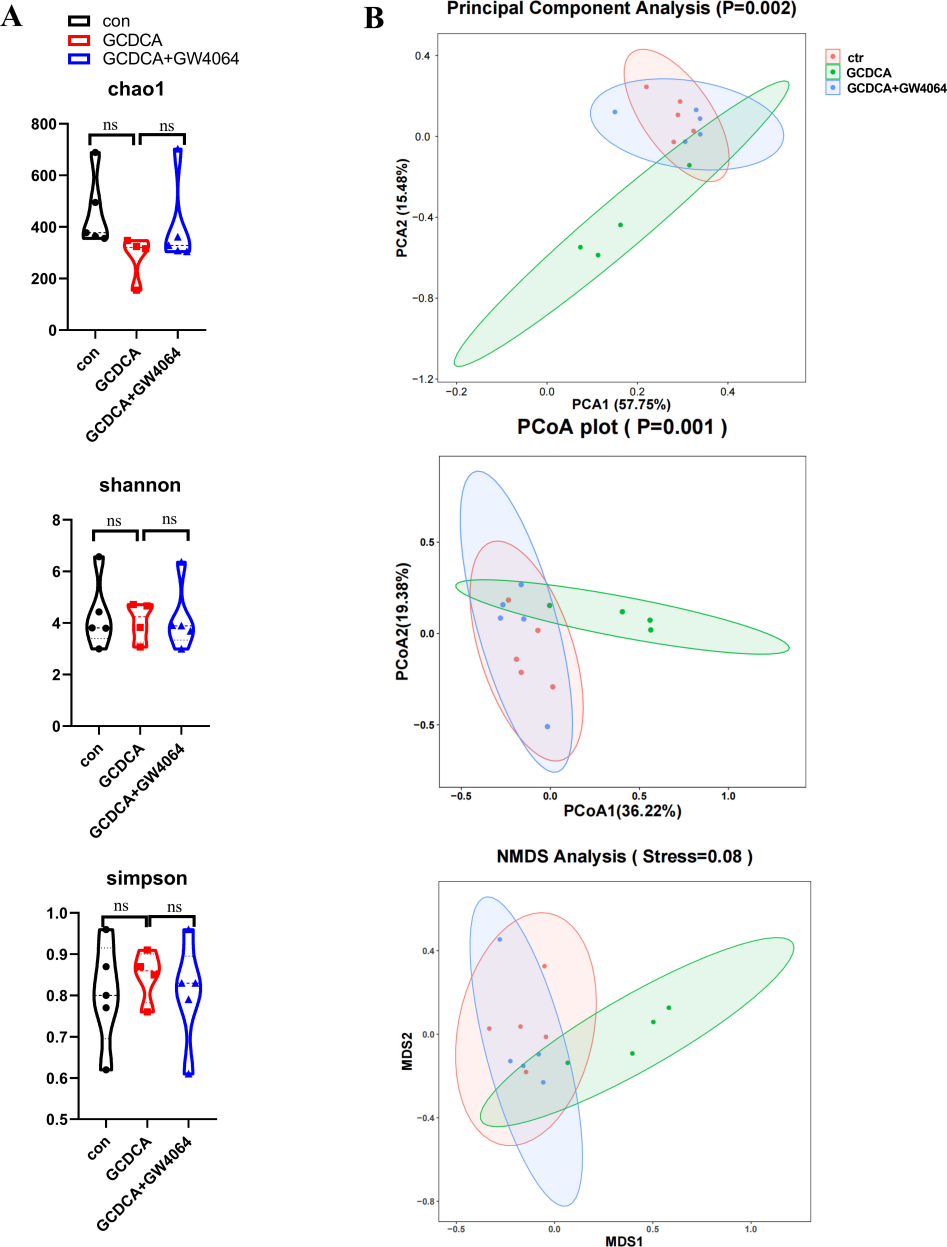
The alpha diversity and beta diversity of microbial community structure in the gut. (**A**) Alpha diversity indices of Chao1, Shannon, and Simpson. (**B**) The beta diversity of microbial community structure: PCA analysis, PCoA (based on Bray-Curtis distances), and NMDS analysis (PCoA based on Bray-Curtis distances).

First, no marked difference in the alpha diversity of the gut microbiome was found among the control, GCDCA, and GCDCA + GW4064 groups on the basis of the Chao1, Shannon, and Simpson indices (Fig. 4A; Supplementary S4). With respect to the beta diversity of the microbial community structure, a PCoA and an NMDS analysis based on Bray-Curtis distances revealed that the GCDCA group was markedly different from the control group and that the GCDCA + GW4064 and control groups had similar microbial communities (Fig. 4B).

**Fig 5:**
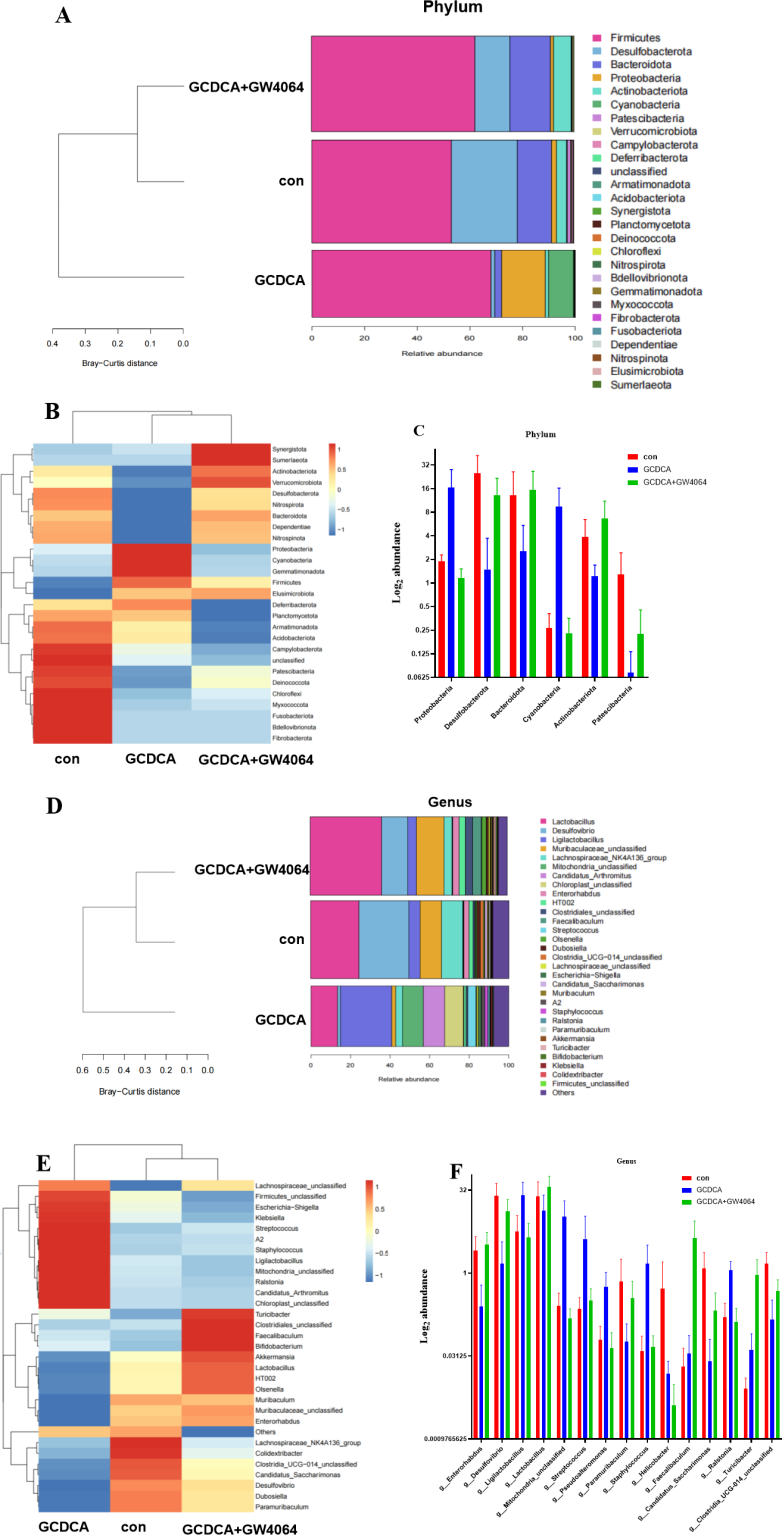
Microbial community structure in phylum and genus levels. (**A**) The structure of microbial community in phylum level among control, GCDCA gavaged, and GCDCA + GW4064 gavaged groups. (**B**) The heatmap of microbial community in phylum level among control, GCDCA gavaged, and GCDCA + GW4064 gavaged groups. (**C**) The remarkable different microbiota in phylum level among control, GCDCA gavaged, and GCDCA + GW4064 gavaged groups. (**D**) The structure of microbial community in genus level among control, GCDCA gavaged, and GCDCA + GW4064 gavaged groups. (**E**) The heatmap of microbial community in genus level among control, GCDCA gavaged, and GCDCA + GW4064 gavaged groups. (**F**) The remarkable different microbiota in genus level among control, GCDCA gavaged, and GCDCA + GW4064 gavaged groups.

At the phylum level, the gut bacteria in the control group, GCDCA group, and GCDCA + GW4064 group consisted of Firmicutes (53.28%, 67.99%, and 62.27%, respectively), Desulfobacterota (25.16%, 1.5%, and 13.28%, respectively), Proteobacteria (1.90%, 16.61%, and 1.17%, respectively), Patescibacteria (1.28%, 0.07%, and 0.22%, respectively), Campylobacterota (0.52%, 0.01%, and 0.0028%, respectively), Bacteroidota (13.08%, 2.55%, and 15.50%, respectively), Actinobacteriota (3.86%, 1.22%, and 6.72%, respectively), and Cyanobacteria (0.27%, 9.52%, and 0.23%, respectively) (Fig. 5A; Supplementary S5). Firmicutes was the main microbiota constituent in the three communities. The abundances of Desulfobacterota, Bacteroidota, and Actinobacteriota were lower in the GCDCA group than in the control group; however, the abundances of Proteobacteria, Cyanobacteria, and Patescibacteria were significantly greater in the GCDCA group than in the control group. Among the three groups, the microbiota structure of the GCDCA group significantly differed from that of the control group at the phylum level, and the microbiota structure of the GCDCA + GW4064 group was similar to that of the control group, as revealed by the Bray-Curtis distances (Fig. 5B and C). Taken together, these findings indicated that GW4064 administration improved the microbiota structure at the phylum level.

At the genus level, the gut bacteria in the control, GCDCA, and GCDCA + GW4064 groups included *Desulfovibrio* (25.16%, 1.48%, and 13.27%, respectively), *Lactobacillus* (24.39%, 13.43%, and 36.25%, respectively), *Ligilactobacillus* (5.63%, 25.93%, and 4.40%, respectively), *Enterorhabdus* (2.58%, 0.25%, and 3.26%, respectively), *Clostridia_UCG-014_unclassified* (1.47%, 0.14%, and 0.48%, respectively), *Candidatus_Saccharimonas* (1.21%, 0.02%, and 0.21%, respectively), *Streptococcus* (0.23%, 4.12%, and 0.32%, respectively), *Paramuribaculum* (0.70%, 0.06%, and 0.35%, respectively), *Mitochondria_unclassified* (0.25%, 10.55%, and 0.15%, respectively), *Helicobacter* (0.52%, 0.01%, and 0.0028%, respectively), *Faecalibaculum* (0.02%, 0.04%, and 4.36%, respectively), *Ralstonia* (0.16%, 1.12%, and 0.13%, respectively), *Turicibacter* (0.01%, 0.04%, and 0.93%, respectively), *Methyloversatilis* (0.04%, 0.19%, and 0.04%, respectively), *Staphylococcus* (0.04%, 1.49%, and 0.05%, respectively), and *Pseudoalteromonas* (0.06%, 0.56%, and 0.04%, respectively) (Fig. 5D; Supplementary S6). The GCDCA group exhibited markedly decreased abundances of *Desulfovibrio*, *Lactobacillus*, *Enterorhabdus*, *Clostridia_UCG-014_unclassified*, *Candidatus_Saccharimonas*, *Paramuribaculum,* and *Helicobacter* as well as markedly increased abundances of *Ligilactobacillus*, *Mitochondria_unclassified*, *Pseudoalteromonas*, *Streptococcus*, *Staphylococcus*, and *Ralstonia* compared with the control group. Moreover, GW4064 restored the abundance of these microbiota at the genus level. Among the three groups, the microbiota structure of the GCDCA group was markedly different from that of the control group, and the microbiota structure of the GCDCA + GW4064 group was similar to that of the control group (Fig. 5E and F). GW4064 administration mitigated the imbalance of the microbiota at the genus level.

**Fig 6:**
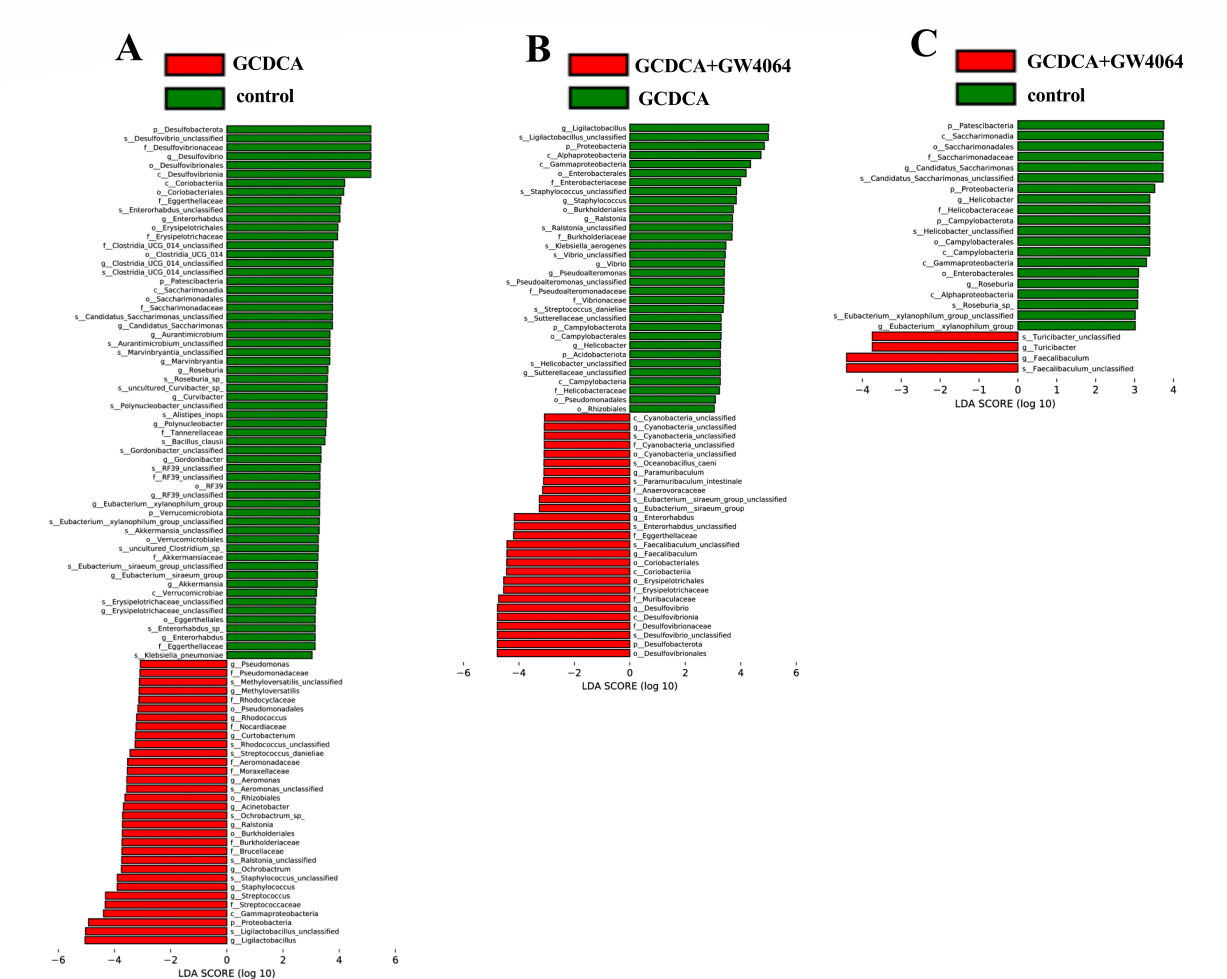
The results of LEfSe analysis displayed the changed gut microbiota structure. (**A**) Microbiota with significant differences between control and GCDCA gavaged groups. (**B**) Microbiota with significant differences between GCDCA gavaged group and GCDCA + GW4064 group. (**C**) Microbiota with significant differences between control and GCDCA + GW4064 groups.

LEfSe analysis results revealed changes in the gut microbiota structure. Ninety-two taxa (containing six grading levels) were shared between the control group and the GCDCA group (Fig. 6A), 59 taxa (containing six grading levels) were shared between the GCDCA group and the GCDCA + GW4064 group (Fig. 6B), and 24 taxa (containing six grading levels) were shared between the control group and the GCDCA + GW4064 group (Fig. 6C). On the basis of the differences in taxa at the six grading levels, the GCDCA group presented markedly altered gut microbiota structure compared with the control group, and the administration of GW4064 alleviated the gut microbiota imbalance caused by BA disorders in the gut.

### Activation of gut FXR protected against GCDCA-induced intestinal injury

**Fig 7:**
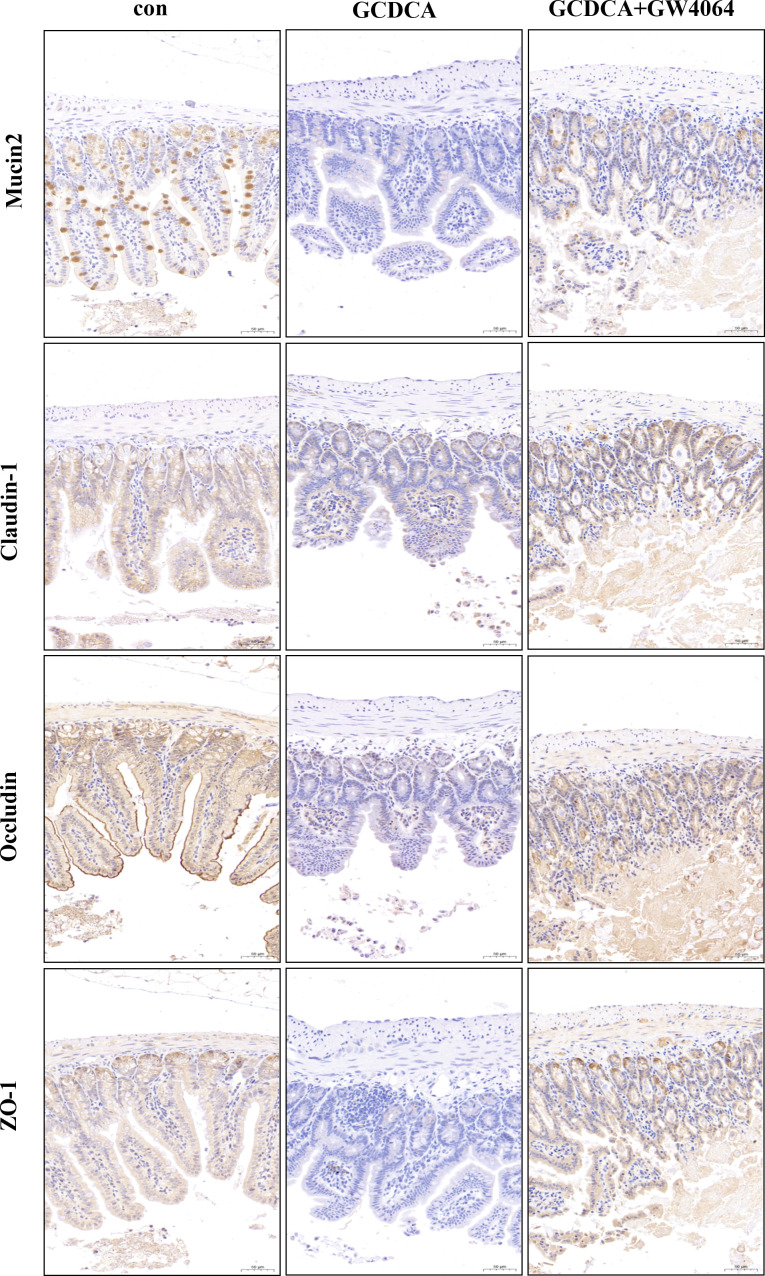
Activation of gut FXR alleviated injury of gut by GCDCA. Immunohistochemistry analysis revealed that mucin2, claudin-1, occludin, and ZO-1 were downregulated in the GCDCA group, whereas GW4064 upregulated their expressions.

To further investigate the effects of GCDCA on the intestinal mucosa, the expressions of mucin2, claudin-1, occludin, and ZO-1 were investigated. Immunohistochemistry analysis revealed that mucin2, claudin-1, occludin, and ZO-1 were downregulated in the GCDCA group, whereas GW4064 upregulated their expression (Fig. 7). Figure 3 also indicates that GW4064 activated gut FXR. These results suggested that gut FXR activation alleviated intestinal injury caused by GCDCA.

### Relationship between the gut microbiota and BA metabolism

Spearman correlation analysis was conducted to determine the correlation between gut microbiota constituents that showed significant differences at the phylum and genus levels and the levels of BAs that showed significant differences in the liver and gut based on the following criteria: |*r*| ≥ 0.5 and *P* < 0.05.

### Relationship between the gut microbiota and gut BA metabolism

**Fig 8:**
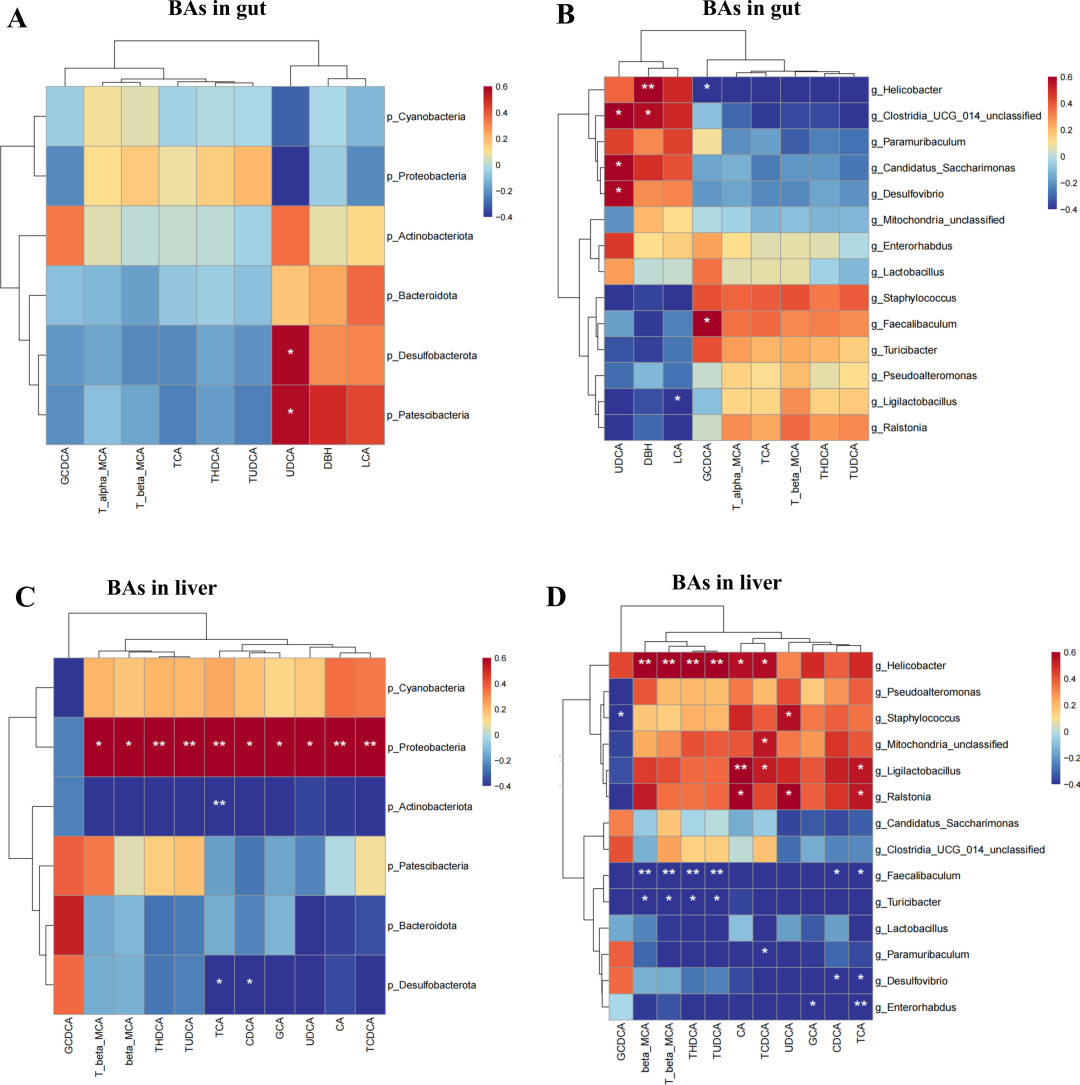
The relationship between the gut microbiota and bile acid metabolism. (**A**) The relationship between the gut microbiota in phylum level and gut bile acid metabolism. (**B**) The relationship between the gut microbiota in genus level and gut bile acid metabolism. (**C**) The relationship between the gut microbiota in phylum level and liver bile acid metabolism. (**D**) The relationship between the gut microbiota in genus level and liver bile acid metabolism.

At the phylum level, the abundances of Desulfobacterota and Patescibacteria were positively related to UDCA levels in the gut (*r* = 0.587, *P* = 0.027; *r* = 0.574, *P* = 0.031, respectively) (Fig. 8A). At the genus level, the abundance of *Helicobacter* was positively related to gut DBH levels (*r* = 0.673, *P* = 0.008) and negatively related to GCDCA (*r* = −0.563, *P* = 0.035). The abundance of *Clostridia_UCG_014_unclassified* was positively correlated with the gut UDCA (*r* = 0.642, *P* = 0.013) and DBH (*r* = 0.571, *P* = 0.032) levels. The abundance of *Faecalibaculum* was positively correlated with the gut GCDCA concentration (*r* = 0.638, *P* = 0.013). The abundance of *Candidatus_Saccharimonas* was positively correlated with the gut UDCA concentration (*r* = 0.599, *P* = 0.023), and the abundance of *Desulfovibrio* was positively correlated with the gut UDCA concentration (*r* = 0.587, *P* = 0.027). The abundance of *Ligilactobacillus* was negatively related to gut LCA levels (*r* = −0.562, *P* = 0.036) (Fig. 8B). These results suggested that some important gut microbes may be involved in the dysregulation of gut BA metabolism caused by GCDCA.

### Relationship between the gut microbiota and liver BA metabolism

At the phylum level, the abundance of Proteobacteria was positively related to the levels of liver TCA (*r* = 0.73, *P* = 0.003), TCDCA (*r* = 0.709, *P* = 0.005), CA (*r* = 0.679, *P* = 0.007), TUDCA (*r* = 0.679, *P* = 0.009), beta-MCA (*r* = 0.67, *P* = 0.01), THDCA (*r* = 0.668, *P* = 0.008), CDCA (*r* = 0.653, *P* = 0.011), T-beta-MCA (*r* = 0.652, *P* = 0.013), GCA (*r* = 0.627, *P* = 0.016), and UDCA (*r* = 0.618, *P* = 0.018). The abundance of Actinobacteria was negatively correlated with the level of liver TCA (*r* = −0.717, *P* = 0.003). The abundance of Desulfobacterota was negatively correlated with the levels of liver CDCA (*r* = −0.552, *P* = 0.04) and TCA (*r* = −0.543, *P* = 0.044) (Fig. 8C).

At the genus level, the abundance of *Faecalibaculum* was negatively related to the levels of beta-MCA (*r* = −0.814, *P* = 0.0003), T-beta-MCA (*r* = −0.677, *P* = 0.007), TUDCA (*r* = −0.677, *P* = 0.007), THDCA (*r* = −0.676, *P* = 0.007), TCA (*r* = −0.658, *P* = 0.01), and CDCA (*r* = −0.598, *P* = 0.023) in the liver. The abundance of *Enterorhabdus* was negatively correlated with the liver levels of TCA (*r* = −0.668, *P* = 0.008) and GCA (*r* = −0.54, *P* = 0.046). The abundance of *Ligilactobacillus* was positively correlated with the levels of CA (*r* = 0.664, *P* = 0.009), TCA (*r* = 0.543, *P* = 0.044) and TCDCA (*r* = 0.538, *P* = 0.049) in the liver. The abundance of *Desulfovibrio* was negatively correlated with the liver levels of CDCA (*r* = −0.552, *P* = 0.04) and TCA (*r* = −0.543, *P* = 0.044) (Fig. 8D). These results suggested that some important gut microbes may be involved in the dysregulated BA metabolism in the liver caused by GCDCA.

## DISCUSSION

Our previous study indicated that GCDCA induces liver fibrosis via the NLRP3 inflammasome pathway in mice and that GW4064 relieves the fibrosis caused by GCDCA (14). Approximately 95% of BAs are reabsorbed in the terminal ileum (18). Therefore, liver/gut BA metabolism and the gut microbiota in the terminal ileum are substantially influenced by the administration of GCDCA via gavage. The current study further investigated the role of GCDCA in BA metabolism and the gut microbiota to help elucidate the role of GCDCA in liver fibrosis.

Several studies have reported that primary BAs are increased and secondary BAs are decreased in the feces of liver fibrosis patients (11, 19), and the level of GCDCA, a primary BA, in the stool of nonobese patients markedly increases with worsening fibrosis severity (12). In the present study, the administration of a high concentration of GCDCA by gavage served as a method for understanding the effects of gut GCDCA on BA metabolism, the intestinal barrier, and the gut microbiota.

T-alpha-MCA and T-beta-MCA, potent FXR antagonists, inhibit FXR activation in the gut and increase BA production in the liver (20). In the present study, high T-alpha-MCA and T-beta-MCA levels in the gut inhibited FXR activation and promoted BA production, whereas GW4064 administration alleviated the effects of T-alpha-MCA and T-beta-MCA. In the present study, the gut TUDCA and THDCA concentrations were high in the GCDCA group. TUDCA alleviates cholestatic liver injury by reducing the BA burden through hepatic FXR activation (21). THDCA attenuates BA production by downregulating CYP7A1 (22). However, the increases in the TUDCA and THDCA levels were markedly lower than the increases in the T-alpha-MCA and T-beta-MCA levels in the present study, and TUDCA and THDCA may not have contributed to these changes.

The total or primary BA levels in the gut of the GCDCA group were greater than those in the control group, but no differences were noted between the GCDCA group and the GCDCA +GW4064 group. With respect to liver BAs, the total or primary BA levels in the GCDCA group were greater than those in the control group, and a marked reduction was observed in the GCDCA + GW4064 group. In the terminal ileum, FXR activation promotes the expression of fibroblast growth factor 19 (human)/15 (mouse), which reaches the liver through the portal vein and inhibits the expression of *Cyp7a1*, attenuating BA production (4, 5). GW4064, an agonist of FXR, activated gut FXR to inhibit BA production in the liver. These results indicated that GCDCA induced cholestasis and that GW4064 inhibited liver BA synthesis.

Figure 3A and B revealed that the increasing trend in total or primary BAs in the liver caused by GCDCA was alleviated by the administration of GW4064. Figure 8C and D show that the gut microbiota, including the phylum Proteobacteria and the genera *Helicobacter*, *Staphylococcus*, *Ligilactobacillus*, *Ralstonia*, *Faecalibaculum*, *Enterorhabdus,* and *Desulfovibrio*, had a marked correlation with liver BA levels under GCDCA intervention. These findings suggest that the gut microbiota is important for liver BA metabolism.

Compared with GCDCA, GW4064 led to an increasing trend in total or primary BA levels in the gut (Fig. 3D and E), suggesting that GW4064 may increase the intestinal excretion of BAs. Figure 5 shows that GW4064 administration alleviated the imbalance in the gut microbiota induced by GCDCA. The results of the correlation analyses revealed positive relationships between the gut microbiota and gut BAs under GCDCA intervention (Fig. 8A and B), suggesting that BA intervention changed the structure of the gut microbiota.

Firmicutes, Bacteroidetes, Proteobacteria, Actinobacteria, Fusobacteria, and Verrucomicrobia are the main members of the human gut microbiota, and Firmicutes and Bacteroidetes account for greater than 90% of the bacteria in the gut (23, 24). Qin and colleagues reported that Bacteroidetes and Firmicutes are the main fecal microbial communities in healthy individuals and liver cirrhosis patients, and that cirrhosis patients have a lower abundance of Bacteroidetes but higher levels of Proteobacteria and Fusobacteria than healthy people do (25). Patients with compensated cirrhosis exhibit a low abundance of Bacteroidetes and a high abundance of Proteobacteria (26, 27). Sung et al. also (27) reported that Firmicutes, Proteobacteria, and Actinobacteria were more abundant in patients with acute hepatic encephalopathy than in patients with compensated cirrhosis. In the present study, the GCDCA group presented greater abundances of the phyla Proteobacteria and Cyanobacteria and lower abundances of Bacteroidota and Actinobacteriota compared with the control group, and the abundances of these phyla returned to baseline levels after the administration of GW4064. A study of the oral microbiome of hepatocellular carcinoma (HCC) revealed a high abundance of Cyanobacteria and cyanobacterial genes, which are involved in the production of microcystin, a hepatotoxic tumor promotor, and exhibited a positive correlation with HCC (28). A study showed that microcystin can result in nonalcoholic fatty liver disease, liver fibrosis, and liver cancer (29). In contrast, the level of the phylum Desulfobacterota and the levels of the genera *Enterorhabdus* and *Desulfovibrio* were increased in the gut of patients with liver fibrosis induced by carbon tetrachloride (CCl4) (30). However, the opposite findings were obtained in this study for the level of the phylum Desulfobacterota and the levels of the genera *Enterorhabdus* and *Desulfovibrio*, suggesting that abnormal BA metabolism caused by GCDCA inhibited the above-mentioned microbes and that the different fibrosis models possess distinct gut microbiota compositions. At the genus level, *Streptococcus* was more abundant in the GCDCA group compared with the control and GCDCA + GW4064 groups. Some studies have reported that patients with cirrhosis and hepatic encephalopathy possess a greater abundance of *Streptococcus* (25, 26). In the present study, *Ralstonia* was detected at greater abundance in the GCDCA group than in the control and GCDCA + GW4064 groups. *Ralstonia* ubiquitously exists in the natural environment, and some species of this genus are involved in plant and crop spoilage (31). *Ralstonia* is more common in patients with hepatic encephalopathy than in patients with cirrhosis (26), and another study revealed that *Ralstonia* is related to impaired lung function in patients with cystic fibrosis (32). These results suggested that high GCDCA levels in the gut result in changes in the gut microbiota, which are involved in liver fibrosis development, as observed in a mouse liver fibrosis model.

Intestinal barrier disruption plays an important role in the process of liver fibrosis (33). Therefore, the gene expression levels of mucin2 and the tight junction-related proteins claudin-1, occludin, and ZO-1 were measured in the terminal ileum. In the present study, GCDCA downregulated mucin2, claudin-1, occludin, and ZO-1 expressions, whereas gut FXR activation restored their expressions. These findings suggested that gut FXR activation improved intestinal injury caused by GCDCA.

### Conclusion

GCDCA induced cholestasis and disturbed BA metabolism in the gut and liver, as well as the intestinal barrier and structure of the gut microbiota. Activation of gut FXR improved intestinal barrier injury and alleviated BA metabolism dysfunction and dysbacteriosis caused by GCDCA under cholestatic conditions.

## Data Availability

The data generated and/or analyzed are available from the corresponding author on reasonable request.

## SUPPLEMENTAL MATERIAL

Supplementary S1 spectrum.03150-24-s0001.docx Detailed LC-MS/MS MRM parameters for bile acid compounds.

Supplementary S2 spectrum.03150-24-s0002.xlsx BAs profile of liver.

Supplementary S3 spectrum.03150-24-s0003.xlsx BAs profile of terminal ileum.

Supplementary S4 spectrum.03150-24-s0004.xlsx Alpha diversity indices of Chao1, Shannon, and Simpson.

Supplementary S5 spectrum.03150-24-s0005.xlsx The Microbial community structure in Phylum level among control group, GCDCA gavaged group, and GCDCA+GW4064 group.

Supplementary S6 spectrum.03150-24-s0006.xlsx The Microbial community structure in Genus level among control group, GCDCA gavaged group, and GCDCA+GW4064 group.

ASM does not own the copyrights to Supplemental Material that may be linked to, or accessed through, an article. The authors have granted ASM a non-exclusive, world-wide license to publish the Supplemental Material files. Please contact the corresponding author directly for reuse.
