# Supplementary material for: Activation of gut FXR improves the metabolism of bile acids, intestinal barrier, and microbiota under cholestatic condition caused by GCDCA in mice
Source: Microbiol Spectr. 2025 Feb 21;13(4):e03150-24. doi: 10.1128/spectrum.03150-24 (PMC11960106; doi:10.1128/spectrum.03150-24)
Supplement: Supplementary S1 — Detailed LC-MS/MS MRM parameters for bile acid compounds. [file spectrum.03150-24-s0001.docx]

Supplementary S1 Detailed LC-MS/MS MRM parameters for bile acid compounds

Table1 The list of reagents

| Chemical  name | CAS | Brand | Pureness |
| --- | --- | --- | --- |
| methanol | 67-56-1 | CNW | LC-MS |
| acetonitrile | 75-05-8 | CNW | LC-MS |
| ammonium acetate | 631-61-8 | CNW | LC-MS |

Table 2 Laboratory equipment

| Equipment | Model | Brand |
| --- | --- | --- |
| Liquid chromatography tandem mass spectrometer | liquid-phase system：Vanquish  mass spectrum：TSQ Altis | Thermo Fisher |
| centrifuge | Centrifuge 5424 R | Eppendorf |
| Frozen grinder | JXFSTPR-CLN | Shanghai Jingxin Industrial Development co., LTD |
| Multifunctional mixer | BCM2500 | Biocomma |

Table 3. Detailed LC-MS/MS MRM parameters for bile acid compounds

| Chemical  name | CAS | Precursor (m/z)  Q1 | Product (m/z)  Q3 | Collision Energy (V) | RF Lens (V) | Adduct Ion | Linear range（ng/mL） | Calibration curve | Coefficient of  determination  （R2 ) | Vender |
| --- | --- | --- | --- | --- | --- | --- | --- | --- | --- | --- |
| DBH | 490-79-9 | 153 | 108.125 | 21.18 | 39 | [M-H]- | 0.500~10.0 | y=-2364.07+24106.3*x | 0.9972 | ZZSTANDARD |
| GDHCA | 3415-45-0 | 458.262 | 74.125 | 30.57 | 90 | [M-H]- | 5.00~50.0 | y=359.409+2515.74*x | 1.0000 | Altascientific |
| 7,12-DKLCA | 517-33-9 | 403.3 | 385.321 | 26.23 | 90 | [M-H]- | 10.0~200 | y=24041+3681.98*x | 0.9996 | Altascientific |
| DHCA | 81-23-2 | 401.35 | 331.292 | 24.84 | 87 | [M-H]- | 5.00~50.0 | y=82.2411+3740.33*x | 0.9990 | ZZSTANDARD |
| UCA | 2955-27-3 | 407.25 | 343.292 | 33.6 | 107 | [M-H]- | 5.00~200 | y=13102.3+1827.69*x | 0.9987 | ZZSTANDARD |
| α-MCA | 2393-58-0 | 407.3 | 405.304 | 29.47 | 99 | [M-H]- | 20.0~1000 | y=168284+4376.09*x | 0.9994 | ZZSTANDARD |
| T-α-MCA | 25613-05-2 | 514.25 | 80.042 | 54.92 | 209 | [M-H]- | 20.0~500 | y = -2408.85+428.372*x | 0.9988 | ZZSTANDARD |
| T-β-MCA | 25696-60-0 | 514.3 | 80.042 | 55 | 228 | [M-H]- | 20.0~200 | y = -15306.8+1558.44*x | 0.9952 | ZZSTANDARD |
| GCDCA-3-S | 66874-09-7 | 528.25 | 448.375 | 29.39 | 95 | [M-H]- | 20.0~500 | y=62816.3+5162.71*x | 0.9991 | ZZSTANDARD |
| β-MCA | 2393-59-1 | 407.3 | 407.3 | 1 | 129 | [M-H]- | 20.0~1000 | y=43839.3+3220.75*x | 0.9996 | ZZSTANDARD |
| ω-MCA | 6830-03-1 | 407.3 | 405.304 | 30.02 | 97 | [M-H]- | 20.0~1000 | y=168284+4376.09*x | 0.9994 | ZZSTANDARD |
| GUDCA | 64480-66-6 | 448.35 | 74.054 | 32.67 | 103 | [M-H]- | 1.00~50.0 | y=2965.68+5512.19*x | 0.9883 | ZZSTANDARD |
| GHCA | 32747-08-3 | 464.3 | 74.054 | 33.73 | 94 | [M-H]- | 10.0~200 | y=34720.2+4207*x | 0.9965 | ZZSTANDARD |
| THCA | 32747-07-2 | 514.3 | 80.03 | 55 | 249 | [M-H]- | 5.00~200 | y=7320.33+1868.44*x | 0.9993 | ZZSTANDARD |
| MDCA | 668-49-5 | 391.3 | 391.3 | 1 | 95 | [M-H]- | 2.00~100 | y=3080.33+3797.91*x | 0.9963 | ZZSTANDARD |
| TLCA-3-S | 64936-83-0 | 280.75 | 97.042 | 27.91 | 76 | [M-2H]/2- | 5.00~100 | y=4094.06+7338.52*x | 0.9977 | Sigma-Aldrich |
| GLCA-3-S | 64936-82-9 | 512.288 | 432.375 | 29.43 | 92 | [M-H]- | 5.00~100 | y=21555.4+8707.05*x | 0.9992 | ZZSTANDARD |
| TUDCA | 14605-22-2 | 498.3 | 80.042 | 55 | 115 | [M-H]- | 20.0~500 | y=23123.4+1724.09*x | 0.9993 | ZZSTANDARD |
| GHDCA | 13042-33-6 | 448.35 | 74.083 | 32.13 | 108 | [M-H]- | 5.00~200 | y=18033.8+3528.28*x | 0.9995 | ZZSTANDARD |
| THDCA | 110026-03-4 | 498.3 | 80.042 | 55 | 111 | [M-H]- | 5.00~200 | y=23123.4+1724.09*x | 0.9993 | ZZSTANDARD |
| HCA | 547-75-1 | 407.3 | 407.3 | 1 | 86 | [M-H]- | 20.0~200 | y=10267.1+2016.35*x | 0.9995 | Anpel |
| GCA | 475-31-0 | 464.35 | 74.125 | 34.44 | 92 | [M-H]- | 2.00~50.0 | y=4589.89+3589.44*x | 0.9997 | ZZSTANDARD |
| UDCA | 128-13-2 | 391.3 | 391.3 | 1 | 87 | [M-H]- | 20.0~500 | y=45557.5+3599.06*x | 0.9998 | ZZSTANDARD |
| TCA | 81-24-3 | 514.3 | 80.042 | 55 | 112 | [M-H]- | 20.0~500 | y=4809.93+759.596*x | 0.9999 | ZZSTANDARD |
| HDCA | 83-49-8 | 391.3 | 391.3 | 1 | 93 | [M-H]- | 5.00~50.0 | y=-13115.7+6531.96*x | 0.9971 | ZZSTANDARD |
| 3α-H-7-0-5β-CA | 4651-67-6 | 389.3 | 389.3 | 1 | 90 | [M-H]- | 20.0~200 | y=78747+5717.54*x | 0.9945 | Anpel |
| CA | 81-25-4 | 407.3 | 289.292 | 38.02 | 96 | [M-H]- | 5.00~50.0 | y=-13552.2+5556.61*x | 0.9990 | ZZSTANDARD |
| ACA | 2464-18-8 | 407.3 | 361.304 | 31.16 | 96 | [M-H]- | 5.00~50.0 | y=-9589+8789.91*x | 0.9973 | ZZSTANDARD |
| 12-KDCA | 5130-29-0 | 389.3 | 389.3 | 1 | 94 | [M-H]- | 5.00~50.0 | y=-9899.72+7840.5*x | 0.9947 | Anpel |
| LCA-3-S | 34669-57-3 | 455.4 | 97.042 | 44.13 | 126 | [M-H]- | 2.00~50.0 | y=-16109+38187.4*x | 0.9973 | ZZSTANDARD |
| NorDCA | 53608-86-9 | 377.288 | 377.288 | 1 | 93 | [M-H]- | 2.00~50.0 | y=-2678.56+8121.33*x | 0.9990 | Altascientific |
| GCDCA | 640-79-9 | 448.3 | 74.054 | 33.01 | 91 | [M-H]- | 1.00~50.0 | y=2248.18+9669.35*x | 0.9991 | ZZSTANDARD |
| TCDCA | 516-35-8 | 498.3 | 124 | 52.35 | 117 | [M-H]- | 10.0~200 | y=12992.3+2345.61*x | 0.9981 | ZZSTANDARD |
| ApoCA | 641-81-6 | 389.312 | 389.312 | 1 | 97 | [M-H]- | 2.00~50.0 | y=8226.47+11005*x | 0.9983 | ZZSTANDARD |
| GDCA | 360-65-6 | 448.325 | 74.054 | 32.76 | 92 | [M-H]- | 1.00~50.0 | y=-4623.68+11972.4*x | 0.9994 | ZZSTANDARD |
| CDCA | 474-25-9 | 391.3 | 391.3 | 1 | 102 | [M-H]- | 2.00~50.0 | y=-2258.01+12951.5*x | 1.0000 | ZZSTANDARD |
| GLCA | 474-74-8 | 432.338 | 74.125 | 31.66 | 86 | [M-H]- | 1.00~20.0 | y=398.201+26271.6*x | 0.9992 | ZZSTANDARD |
| TLCA | 6042-32-6 | 482.35 | 80.012 | 55 | 107 | [M-H]- | 1.00~20.0 | y=793.372+10416.6*x | 0.9998 | ZZSTANDARD |
| DCA | 83-44-3 | 391.288 | 345.304 | 32.46 | 89 | [M-H]- | 1.00~50.0 | y=4259.49+32955.4*x | 1.0000 | ZZSTANDARD |
| isoalloLCA | 2276-93-9 | 375.3 | 375.3 | 1 | 91 | [M-H]- | 5.00~50.0 | y=7602.55+21170*x | 0.9998 | Altascientific |
| ILCA | 1534-35-6 | 375.3 | 375.3 | 1 | 93 | [M-H]- | 5.00~100 | y=39802+19727*x | 0.9951 | ZZSTANDARD |
| IDCA | 566-17-6 | 391.3 | 345.304 | 31.75 | 94 | [M-H]- | 1.00~20.0 | y=11717.6+84021.2*x | 1.0000 | ZZSTANDARD |
| OCA | 459789-99-2 | 419.338 | 419.338 | 1 | 105 | [M-H]- | 5.00~100 | y=21389.2+16161.6*x | 0.9994 | ZZSTANDARD |
| LCA | 434-13-9 | 375.3 | 375.3 | 1 | 93 | [M-H]- | 1.00~20.0 | y=9728.54+34964.6*x | 0.9997 | ZZSTANDARD |
